# Approaches to Learning of Preschool Children in China: A Comparison between Only Children and Non-Only Children

**DOI:** 10.3390/bs13050418

**Published:** 2023-05-15

**Authors:** Xiumin Hong, Qianqian Liu, Sijie Zhao

**Affiliations:** Faculty of Education, Beijing Normal University, Beijing 100875, China

**Keywords:** approaches to learning, only children, non-only children, latent profile analysis

## Abstract

Preschool children are at the initial stage of individual development and at a critical period in cultivating their approaches to learning. In the context of China’s frequently adjusted birth policies, further research is necessary on children’s approaches to learning in families of different sizes. A questionnaire survey was administered to 5454 only child and 4632 non-only child parents from the east, middle, and west of China. The study found that children’s approaches to learning generally developed well, but non-only children’s approaches to learning was significantly lower than those of only children. There are four profiles of approaches to learning for both the only children and the non-only children. This study also found that gender, social skills, family income, and type of preschool exert significant influences on children’s approaches to learning. Parents’ educational background had a significant influence on only children’s approaches to learning but had no significant influence on non-only children’s approaches to learning. We provide practical implications for promoting children’s approaches to learning in families of different sizes.

## 1. Introduction

As more attention is paid to the sustainable and lifelong development of individuals around the world, researchers have gradually shifted from the perspective of ‘what young children learn’ to ‘how young children learn’ [1,2,3]. In this context, the term ‘approaches to learning’ has attracted extensive attention from researchers in China and abroad. Rather than referring to specific knowledge and skills, approaches to learning comprise a series of abilities that children need to participate in learning and achieve learning goals [4,5]. Approaches to learning play a fundamental role in children’s early and lifelong development [6,7,8]. They provide a foundation to support preschool children’s all-round development and have been comprehensively considered by foreign policy makers and researchers.

To address the country’s aging population, China has repeatedly adjusted its fertility policy in recent years. China’s fertility policy has undergone tremendous changes since 1978. Since 1978, China has had a one-child policy, which changed to universal two-child policy, and then to the three-child policy. On 31 May 2021, China proposed further optimization of the country’s birth policy and implemented a policy whereby a couple could have three children [9]. The difference in family size is reflected not only in the number of children but also in children’s living environments and educational placements. Different family environments (i.e., only children families vs. non-only children families) may significantly impact children’s development of their approaches to learning. How do preschool children’s approaches to learning develop in families of different sizes? What key factors influence the development of preschool children’s approaches to learning? Focusing on preschool children’s current approaches to learning and the factors influencing them, we set out to provide empirical references for the development of preschool children’s approaches to learning in families of different sizes.

## 2. Literature Review

### 2.1. Differences in Development of China’s Only Children and Non-Only Children

Children’s social and family environments have changed dramatically with the changes in fertility policy. The three-child policy has meant that some families now have more children. Moving from one- to two- or three-child families has brought about great changes in the number of children and the ecological environment in which children grow up. There are significant differences in parenting styles, parenting effectiveness, and parent–child interaction and contact in only children and non-only children families. As a result, there may be differences in children’s approaches to learning in families of different sizes. In addition, the development characteristics of children in different-sized families have always been a contentious issue. We need to understand the differences in children’s approaches to learning to promote these approaches in different-sized families.

Researchers have found significant differences in the physical health, language, and social-emotional development of only and non-only children. However, no consensus exists on whether only children or non-only children achieve more positive outcomes in development of these aspects. For example, the quantity-quality tradeoff held that the quality of children’s development decreases with the increase in number of siblings [10]. The resources dilution model demonstrated that parental resources are limited, and newly born siblings reduce the resources available for older children [11]. Therefore, as the number of siblings rises, the level of children’s access to education declines. Empirical research shows that, in terms of language, only children have better verbal memory than non-only ones [12]. However, some studies found that the health and social development of only children were less optimal than those of non-only ones [13,14]. These conflicting theories and research findings prompted our investigation of differences in children’s development in different family sizes. In particular, differences between only children and non-only children’s approaches to learning remain unclear.

### 2.2. Current Knowledge of Approaches to Learning

Approaches to learning’ is an umbrella term encompassing a broad set of learning-related skills that reflect children’s enthusiasm for and engagement in educational activities [15,16]. The approaches reflect non-intellectual factors involved in learning activities—mainly learning motivation, attention, persistence, and attitude toward learning [17]. Compared with specific knowledge, the approaches to learning emphasize preschool children’s typical responses to people, objects, or experiences in different situations; that is, how children acquire and apply knowledge skills. These learning approaches are more closely related to preschool children’s academic achievements and future development, and they are key predictors of their learning and social development [8,18]. Compared with factors such as intelligence that are not easy to change, approaches to learning can be shaped and changed [17,19]. Research shows that children with positive approaches to learning participate more readily in learning activities and are more likely to experience academic success in the future [20,21]. Therefore, paying attention to children’s approaches to learning is particularly crucial.

Researchers have carried out extensive research into children’s approaches to learning. Vitiello et al. (2011) found children’s competence motivation, attention/persistence, and attitudes toward learning were at a medium to high level for 191 children of 3–5 years of age in the United States [22]. A study of 437 preschool children in China found that the approaches to learning of Chinese children were above average, with scores ranging from high to low for competency motivation, learning strategies, concentration, and perseverance [23]. Another study of young Chinese children also found that the preschool children’s approaches to learning development were above average but uneven in different aspects of development [24]. In addition, Angelo (2006) investigated 1665 children aged 3–6 years in his Head Start Child Outcomes Framework, finding that children’s approaches to learning could be divided into several types. These included ‘excellent,’ with good attention and attitude toward learning, children with good learning motivation, and those with poor learning motivation [25]. Buek (2019) tracked 18,174 children from preschool to the second grade of primary school, finding that children could be differentiated by approaches to learning (high vs. lower levels) at the kindergarten stage [26]. This indicates the heterogeneity of children’s approaches to learning. According to our research experience, Chinese researchers tend to investigate the mean value of children’s approaches to learning but ignore the group differences in approaches to learning. In other words, it is necessary to examine the latent categories of approaches to learning of Chinese children.

Person-centered analysis divides individuals with similar patterns into one category based on a set of indicators [27] and allows for the identification of profiles of individuals presenting distinct configurations of these personality characteristics [28], which is especially suitable for situations where structural differences may be unevenly distributed. Therefore, this study adopted the latent profile analysis (LPA), a person-centered analytic strategy.

### 2.3. Factors Influencing Preschool Children’s Approaches to Learning

Preschool children’s approaches to learning are affected by individuals, families, and preschools in their development process. At the individual level, some researchers found that girls’ approaches to learning were at a significantly higher level than boys’ [29]. However, some studies reported no significant difference in preschool children’s approaches to learning in terms of their genders. In addition, researchers found that preschool children’s social skills affected their approaches to learning. Bulotsky-Shearer et al. (2011) demonstrated that children’s social skills in specific situations (e.g., not participating in activities, being unable to regulate their own behaviors, and lacking exposure to social events) were negatively correlated with their approaches to learning [30]. Social performance in interaction with peers and teachers was also important in influencing less favorable approaches to learning. Fantuzzo (2005) found that early aggressive behavior could be used to predict poorer learning attitudes, and inattention predicted lower competence motivation, attention, and persistence in learning tasks [31].

In addition to individual factors, family-related aspects, such as family income and parents’ educational background, are associated with the development of children’s approaches to learning. Gershoff (2003) found that higher family income was associated with more optimal approaches to learning in children, such as involving self-regulation, when they graduated from preschool [32]. Bundy (2006) found that the mother’s education level could be used to explain 2% of the variance of approaches to learning [33]. Children whose mothers had graduate degrees had the highest average scores for approaches to learning.

At the preschool level, the type of preschool may be associated with children’s approaches to learning. In China, obvious differences in the quality of education are found between public and private preschools [34]. Differences in teachers, educational concepts, and curriculum settings are observed between public and private preschools, with better approaches to learning in children in public preschools [35].

**Figure 1:**
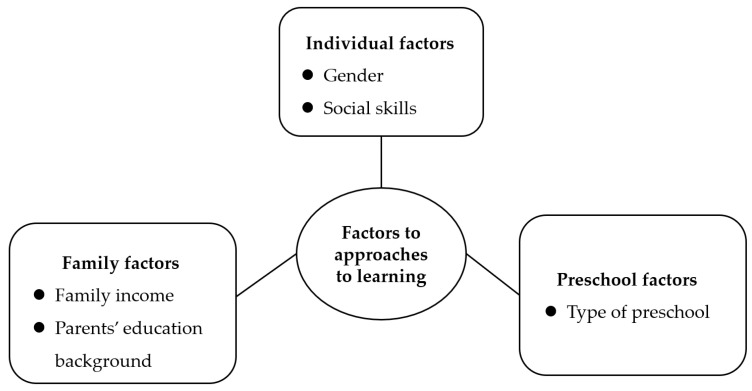
Theoretical framework of the study.

To more specifically promote children’s approaches to learning in families of different sizes, this study focused on factors influencing only and non-only children’s approaches to learning (Figure 1).

### 2.4. The Present Study

After sorting out the existing relevant studies, it can be found that the current research has the following research space: First, although researchers have paid attention to the approaches to learning of preschool children, few studies have paid attention to the differences in the approaches to learning of preschool children with different family sizes. Under the social background of updating population policy in China, it is of practical significance to pay attention to the differences in the approaches to learning of children in different family sizes in China. Second, existing studies, especially those in China, pay more attention to the mean value of approaches to learning, while few studies focus on the development types of approaches to learning. Third, the existing studies on the influencing factors of approaches to learning are mostly analyzed from a certain aspect, and few are comprehensively analyzed from the three aspects of individual, family, and preschool.

On the basis of the three-child policy, this study investigated the development of children’s approaches to learning in different family sizes and the effects of individuals (gender and social skills), family (family income and parental education background), and preschool (type of preschool) on children’s approaches to learning. The study aimed to provide an evidence-based reference for improving children’s approaches to learning in families of different sizes. The following research questions were explored:

(a) Is there a difference in approaches to learning between only children and non-only children?
(b) How many latent profiles are there in the approaches to learning between only children and non-only children?
(c) What factors influence the approaches to learning categories of only children and non-only children?

## 3. Materials and Methods

### 3.1. Participants

A stratified convenience sampling was used in this study. Sampling was carried out in two cities selected based on a comprehensive consideration of economic development level, in each of two provinces; respectively, in the east, middle, and west regions of China. Preschools were selected depending on the education departments of each province and city. In this study, the survey was administered to parents of preschool children in 12 cities, including Qingdao in Shandong, Zhengzhou in Henan, and Chengdu in Sichuan. A total of 10,086 parents were included as participants in the study.

The participants were parents of 5454 only children and 4632 non-only children. In the only-child group, preschool children in junior, middle, and senior classes accounted for 29.5%, 38.9%, and 31.6%, respectively, and boys and girls for 56.0% and 44.0%, respectively. Preschool children with family income less than 5000 yuan comprised 33.7% of the sample, 5000 to 12,000 yuan was the income bracket for 49.2% of the sample, and families earning more than 12,000 yuan made up 17.1%. Children in public preschools and private preschools accounted for 84.6% and 15.4%, respectively. In the non-only-child group, children in junior, middle, and senior classes accounted for 27.6%, 35.5%, and 36.9%, respectively, and boys and girls for 50.3% and 49.7%, respectively. The preschool children whose family incomes were less than 5000 yuan, 5000 to 12,000 yuan, and more than 12,000 yuan, made up 51.9%, 37.5%, and 10.6% of the sample, respectively. As for the only children, most of the non-only children (84.3%) attended public preschools and private preschools and accounted for 15.7%.

### 3.2. Measurement

#### 3.2.1. Preschool Learning Behaviors Scale

The preschool learning behaviors scale (PLBS) has been used to investigate the approaches to learning of preschool children in many countries [17]. Similarly, in China, this scale is often used to investigate the approaches to learning of preschool children, and it has high reliability and validity in the Chinese cultural context. This study used the revised version [36], adapted to ensure its applicability to preschool children’s parents in China. Twenty-five items are in the revised PLBS: three dimensions of competence motivation (e.g., ‘Accepts new activity without fear/resistance’, 10 items); attention/persistence (e.g., ‘Sticks to activity as expected for age’, 8 items); and learning strategy (e.g., ‘Charms others to do work’, 7 items). A three-point scale was used (0 = Most often applies, 1 = Sometimes applies, 2 = Does not apply). In this study, Cronbach’s α coefficients of the dimensions reported by parents of preschool children were 0.84, 0.84, and 0.81, respectively.

#### 3.2.2. Preschool and Kindergarten Behavior Scale

The preschool and kindergarten behavior scale (PKBS) was used to investigate preschool children’s social skills [37]. The 34 social skills items are as follows: three dimensions of social cooperation (e.g., ‘Shares toys and other belongings’, 12 items); social interaction (e.g., ‘Seeks comfort from an adult when hurt’, 11 items); and social independence (e.g., ‘Plays with several different children’, 11 items). The scale used a four-point scale (0 = never true, 1 = rarely true, 2 = sometimes true, and 3 = often true). In this study, Cronbach’s α coefficients of the three dimensions reported by parents of preschool children were 0.85, 0.82, and 0.84, respectively.

### 3.3. Procedure

After obtaining permission from the preschools to conduct the study, we selected families to take part in the survey. Parents provided written consent to participate in the study after receiving information about the research objectives and being assured that the information collected would be used solely for research purposes. We asked the preschools to send our e-questionnaires to parents. Each parent received a separate email containing the electronically linked questionnaire and a letter explaining the instructions and rules to be followed. To avoid the subject effect, we did not collect the preschool name, children’s names, and other demographic information that could identify the preschool or child. Altogether, 10,430 questionnaires were returned. We discarded 344 of these questionnaires because the parents had not followed the instructions, which meant we obtained a final sample of 10,086 completed questionnaires; that is, a return rate of 96.70%.

### 3.4. Data Analysis

We obtained descriptive statistics and performed correlation and regression analyses using SPSS version 22. For latent profile analysis, we used Mplus version 7.4. We also compared several fitting indexes among the models to determine the optimal models. Their values were obtained for the Akaike information criterion (AIC), the Bayesian information criterion (BIC), the sample-size adjusted Bayesian information criterion (SSA-BIC), entropy, and Lo-Mendell-Rubin test (LMRT) models. Entropy values were used to evaluate the accuracy of the model classification. Closer approximation of the entropy value to 1 reflected greater accuracy of the model classification. Entropy values ≥ 0.80 indicated that the accuracy of the classification was over 90% [38]. Moreover, a significant *p*-value of the LMRT indicated that the k classes model was significantly better than the k−1 classes model [39].

## 4. Research Results

### 4.1. Development Status of Children’s Approaches to Learning

The overall score of children’s approaches to learning was above the middle level (*M* = 1.45, *SD* = 0.42). There was a difference in the scores for each dimension, and the overall development of children’s approaches to learning was imbalanced. Among the dimensions, learning strategy had the highest score (*M* = 1.48, *SD* = 0.45), followed by competence motivation (*M* = 1.46, *SD* = 0.40). Attention/persistence scores were the lowest (*M* = 1.41, *SD* = 0.48).

To further understand the relationship between children’s approaches to learning development in the two types of families, independent samples *t*-tests were used to determine differences between only children and non-only children in the three dimensions of competence motivation, attention/persistence, and learning strategy, and in the total scores for approaches to learning. The total scores for the above factors and the approaches to learning of non-only children were significantly lower than those of only children (Table 1). Especially in terms of learning strategy, the scores of only children (*M* = 1.52, *SD* = 0.44) were much higher than non-only children (*M* = 1.43, *SD* = 0.45) and the scores of attention/persistence among only children (*M* = 1.44, *SD* = 0.48) were less higher than that of non-only children (*M* = 1.37, *SD* = 0.48).

### 4.2. Latent Profile Analysis of Children’s Approaches to Learning

The analysis of the sample at the variable level reflected the common problems to a certain extent. However, individual differences among samples could not be fully considered. On the basis of this, a latent profile model was established with each dimension of the approaches to learning of only children and non-only children as the explicit indicators. The purpose was to analyze the heterogeneity of the approaches to learning within the only children and non-only children family groups. As shown in Table 2, considering the AIC, BIC declined trend, entropy value, LMRT significance, and model simplicity, four types of models were selected as the final analysis models.

**Figure 2:**
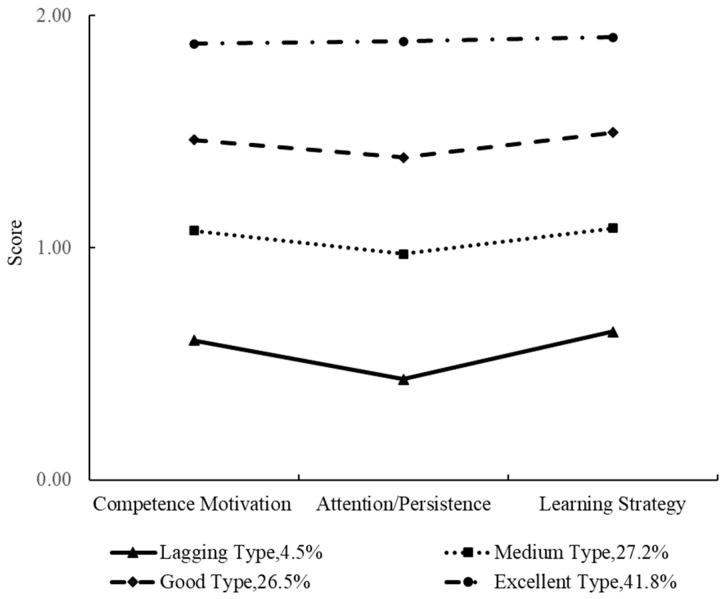
Latent profile analysis of approaches to learning in only children.

**Figure 3:**
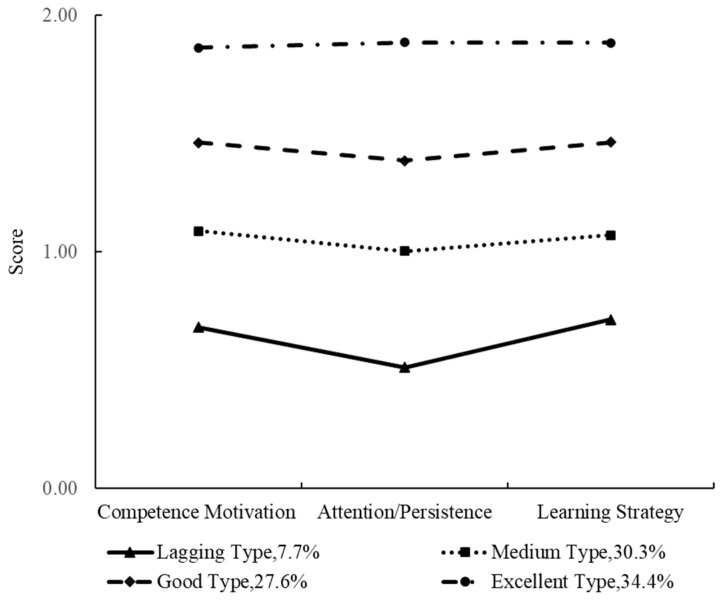
Latent profile analysis of approaches to learning in non-only children.

Figure 2 and Figure 3 show that children in the first group had competence motivation, attention/persistence, and learning strategy scores between 0.43 and 0.72, which were lower than those of the other three approaches to learning types. Therefore, the first type was named the ‘lagging’ type, with 248 only children and 358 non-only children belonging to this group, accounting for 4.5% and 7.7% of the sample, respectively. The three dimensions for the second group’s approaches to learning were between 0.97 and 1.09, which were in the mid-range. This second group was named the ‘medium’ type, and 1484 only children and 1403 non-only children belonged to this type, accounting for 27.2% and 30.3%, respectively. The three dimensions for the third type of children’s approaches to learning were in the range 1.39–1.50, which were higher than the first two types but lower than the fourth. Therefore, this group was named the ‘good’ type, and 1445 (26.5%) only children and 1277 (27.6%) non-only children belonged to this type. For the fourth group, the scores of competence motivation, attention/persistence, and learning strategy were between 1.86 and 1.91, which were higher than those of the other types. They were called the ’excellent’ type and 2277 (41.8%) only children and 1594 (34.4%) non-only children belonged to this group.

### 4.3. Analysis of Factors Influencing Children’s Approaches to Learning

With children’s approaches to learning as the dependent variable, and individual factors (gender and social skills), family factors (family income and parents’ educational background), and preschool factors (type of preschool) as independent variables, the linear regression analysis was conducted to determine how individual, family, and preschool factors are associated with children’s approaches to learning. The regression model results are given below and summarized in Table 3.

#### 4.3.1. Individual Factors

At the individual level, the approaches to learning of only children and non-only children were affected by gender and social skills. Specifically, girls were more inclined to show good approaches to learning than boys, and the approaches to learning of non-only children (*β* = 0.033, *p* < 0.05) were more readily influenced by gender than those of only children (*β* = 0.025, *p* < 0.05). Social skills for only children (*β* = 0.342, *p* < 0.001) and non-only children (*β* = 0.383, *p* < 0.001) had a positive association with the approaches to learning development; that is, higher social skills scores meant better performance in approaches to learning.

#### 4.3.2. Family Factors

Family income was an important family factor affecting children’s approaches to learning. After individual level factors were controlled, family income influenced the approaches to learning development of only children (*β* = 0.096, *p* < 0.001) and non-only children (*β* = 0.155, *p* < 0.001). Higher family income meant better approaches to learning development in children. The educational background of parents is associated with only children’s approaches to learning (*β* = 0.063, *p* < 0.001). Higher parental educational background meant that these children had better approaches to learning. Parents’ educational background had an insignificant influence on approaches to learning of non-only children (*β* = 0.001, *p* > 0.05).

#### 4.3.3. Preschool Factors

Type of preschool was a key influencing factor. After both individual and family factors were controlled, the type of preschool still significantly influenced children’s approaches to learning development. With type of preschool transformed into a dummy variable, analysis revealed that the approaches to learning of children in private preschools tended to be less optimal than for those in public preschools. With each change of one standardized unit for the type of preschool, the approaches to learning of only children decreased by 0.046 standardized units and that of non-only children went down by 0.040 standardized units.

## 5. Discussion

Children are embarking on their individual development trajectories and are in a critical period for cultivating their approaches to learning. Given China’s changing birth policies, more research is needed on children’s approaches to learning development in families of different sizes. This study focused on the differences in the approaches to learning development of only children and non-only children, and on their influencing factors. Parents who participated in this study reported that their children’s approaches to learning are developing well on the whole. However, non-only children’s approaches to learning lag behind only children’s approaches to learning. In addition, this study found that children’s gender, social skills, family income, and the type of preschool are associated with their approaches to learning. Parents’ educational background only has a significant influence on only children, and no significant effect was found on the approaches to learning of non-only children. The results of this study have important implications for devising educational intervention programs for only and non-only children.

### 5.1. Children’s Approaches to Learning Are Developing Well but Non-Only Children’s Approaches to Learning Lag behind Those of Only Children

Approaches to learning refer to children’s tendencies, attitudes, habits, and style in the learning process [2]. The research results show that the average value of children’s approaches to learning in this study was above the mid-level. This is a positive sign, which is most likely linked to the improvement of preschool education quality in China. According to the statistics of the Ministry of Education, China has steadily improved preschool education and significantly increased the number of teachers and their educational levels [40]. Teachers’ understanding of education and educational behavior has been enhanced to promote children’s lifelong learning and development. Thus, teachers are well equipped to consciously cultivate children’s approaches to learning. Considering all the aspects that comprise approaches to learning, children’s approaches to learning develop in an unbalanced way. Learning strategy and competence motivation were reaching high levels in this study, but both attention/persistence developed less well. This may be related to the age characteristics of children. Children have a strong curiosity, a thirst for knowledge, and a strong motivation to participate in activities. However, the excitation and inhibition functions of the cerebral cortex have not yet reached a balance in children, and their rule consciousness has not been internalized, resulting in a lag in the development of attention and persistence.

The results of this study show that only children have significantly better approaches to learning than non-only children. In the context of China’s changing birth policies, the differences in the development of children in families with different child numbers have become the focus of researchers. This study indicated that only children are more likely to develop optimal approaches to learning, conforming to the views of the resource dilution theory [41] and the quantity-quality model [42]. In short, compared with non-only children, only children access more of their parents’ resources, including time, money, and energy. Therefore, they have more external support to promote their approaches to learning, and their approaches to learning become increasingly better.

The approaches to learning of only and non-only children can be divided into four types. The scores of the four types were significantly different for only children and non-only children when considering the three dimensions of the approaches to learning. This finding indicates that children’s approaches to learning are heterogeneous. By analyzing the types of approaches to learning, we found that both only and non-only children who belong to the poor development groups (i.e., lagging type and medium type) comprise 31.7% and 38.0%, respectively. This shows that, although the average value of children’s approaches to learning is above the middle level, more than 30% of children still lag behind, suggesting a need to improve children’s approaches to learning in China.

### 5.2. Children’s Approaches to Learning Are Affected by Individual, Family, and Preschool Factors

At the individual level, the results showed that children’s approaches to learning in different family sizes are affected by gender. The influence of gender on children’s approaches to learning is not affected by the number of siblings. The consistent finding is that girls have stronger learning motivation, better attention and persistence, and a sounder learning strategy than boys. This is consistent with other Chinese research results [29]. The finding may be related to the earlier and better development characteristics of individual girls. In addition, girls are often quieter than boys, leading to advantages in the development of attention and persistence. Moreover, this study found that social skills significantly affected the approaches to learning development of only and non-only children. This is consistent with previous findings that approaches to learning are positively correlated with social skills [43]. This result may be because children who interact well with parents, teachers, and other children are more likely to receive guidance and help in the learning process, leading to continual advancement beyond their own original approaches to learning, stronger learning motivation and attention/persistence, and mastery of their learning strategies.

At the family level, this study found that family income can affect children’s approaches to learning development. According to the family stress model, higher family income means more family education capital and resources for children [44,45], irrespective of whether they are only or non-only children. In other words, parents with high family income are more likely to purchase appropriate learning materials (e.g., educational toys and picture books) and provide opportunities for their children to participate in stimulating activities. These aspects contribute to children’s development of approaches to learning. The results of this study show that the positive influence of parental educational background is limited to only children’s approaches to learning. Parental education status indicates the quality of parents’ family education and parent–child interaction to a certain extent. Only children are sensitive to the parents’ child-rearing behavior. However, in non-only children, siblings are also available for interaction, meaning children are less affected by the quality of parenting because of the other opportunities for interaction with siblings.

We found that children with public preschool education experience are more likely to lag behind than those who attend private preschools; this finding held true for only and non-only children. There are differences in education quality between the private and public preschools in China. Private preschools are run mostly for profit, catering to parents’ needs to boost their children’s abilities and often emphasizing the one-sided connection of explicit knowledge while ignoring the cultivation of children’s competence motivation and learning habits. In contrast, the quality of education in public preschools is uniformly high because teachers are of a relatively high professional level, have scientific knowledge of educational concepts and methods, and pay more attention to the cultivation of children’s approaches to learning.

### 5.3. Practical Implications and Limitations

The current findings have practical implications for understanding and improving young children’s approaches to learning. First, early educators can promote the development of approaches to learning of children, especially non-only children, guide the development of attention and perseverance, and help children learn to adopt more flexible learning strategies in tasks. Second, for young children lagging behind in approaches to learning—especially boys and those with delayed social skills—individualized education through intervention programs should be considered to help children achieve acceptable levels of approaches to learning [46]. Third, preschool teachers should establish a warm and supportive relationship with children [46,47,48], and the government could provide private preschool teachers with more training to effectively cultivate children’s approaches to learning. Fourth, because the development of non-only children’s approaches to learning is also affected by their parents’ educational background, social policies should be formulated to prevent poor approaches to learning in only children of parents with lower education levels [49]. The government could boost children’s approaches to learning by organizing parenting training under the instruction of education experts. This training could improve parenting skills [50], help parents grasp the features of children’s learning, enhance their perception to approaches to learning, and promote parents’ active participation in creating a constructive learning environment for their children [51].

The current study had certain limitations. First, only questionnaires were used for data collection because we were required to maintain social distance because of the COVID-19 pandemic. Future studies could collect more objective data, such as interviews and observations of children. Second, our study did not consider all diverse characteristics of children (e.g., race/ethnicity and residential area). China’s vast territory and the imbalance in young children’s living contexts suggest it would be helpful to include more diverse samples together with detailed background information about participants. Third, future research could introduce other external variables, such as parenting styles, parent involvement, and home-kindergarten cooperation, into the model to gain a more comprehensive understanding of the factors influencing children’s approaches to learning [49,52].

## 6. Conclusions

Cultivating young children’s approaches to learning is of fundamental benefit to their later development. Further research on the development of children’s approaches to learning in different family contexts is necessary in the context of continuous adjustments to China’s fertility policy. This study focused on the developmental differences in approaches to learning between only and non-only children. The study found that children’s approaches to learning were developing well on the whole, but non-only children lag behind only children. Four heterogeneous types of approaches to learning were documented for only and non-only children. In addition, this study confirmed that gender, social skills, family income, and preschool type are associated with children’s approaches to learning. Parental education background has a unique and significant influence on approaches to learning of only children. The findings contribute to more targeted suggestions for the cultivation of approaches to learning in children from families of different sizes.

## Figures and Tables

**Table 1:** An examination of differences in approaches to learning between only children and non-only children.

Variables	Only Children (*n* = 5454)		Non-Only Children (*n* = 4632)		*t*
	*Mean*	*SD*	*Mean*	*SD*	
Competence motivation	1.49	0.40	1.43	0.40	8.088 ***
Attention/persistence	1.44	0.48	1.37	0.48	7.036 ***
Learning strategy	1.52	0.44	1.43	0.45	9.527 ***
Approaches to learning	1.48	0.42	1.41	0.42	8.641 ***

*Note:* *** *p* < 0.001 (two-tailed).

**Table 2:** Model fit indicators of the latent profile of children’s preschool readjustment in different risk regions.

Model	AIC	BIC	SSA-BIC	Entropy	LMRT (*p*)	Latent Profile Proportions
Only children (*n* = 5454)						
2	8685.875	8751.916	8720.139	0.906	10,733.241 ***	0.441, 0.559
3	5052.355	5144.813	5100.325	0.893	3538.699 ***	0.236, 0.315, 0.449
4	2529.710	2648.584	2591.385	0.918	2459.192 ***	0.272, 0.045, 0.265, 0.418
5	1527.319	1672.609	1602.700	0.892	981.862 **	0.201, 0.200, 0.035, 0.373, 0.190
Non-only children (*n* = 4632)						
2	7668.297	7732.704	7700.928	0.895	8673.388 ***	0.502, 0.498
3	4446.469	4536.639	4492.152	0.883	3136.918 ***	0.352, 0.270, 0.378
4	2312.693	2428.627	2371.429	0.904	2080.165 ***	0.077, 0.303, 0.276, 0.344
5	1453.177	1594.873	1524.966	0.884	842.561 *	0.231, 0.055, 0.229, 0.305, 0.180

*Note:* * *p* < 0.05, ** *p* < 0.01, *** *p* < 0.001; AIC = Akaike information criterion, BIC = Bayesian information criterion, SSA-BIC = sample-size Adjusted BIC, LMRT = Lo-Mendell-Rubin test.

**Table 3:** Summary of multivariate logistic regression on the factors influencing children’s preschool readjustment profiles by risk region.

Variable	Approaches to Learning	
	Only Children	Non-Only Children
**Individual factors**		
Gender	0.025 *	0.033 *
Social skills	0.342 ***	0.383 ***
**Family factors**		
Family income	0.096 ***	0.155 ***
Parents’ education background	0.063 ***	0.001
**Kindergarten factors**		
Type of preschool	−0.046 ***	−0.040 **
*R* ^2^	0.159	0.195
Adjusted *R*^2^	0.158	0.194
*F* Value	204.452 ***	222.787 ***

*Note:* * *p* < 0.05, ** *p* < 0.01, *** *p* < 0.001; ‘Gender’ and ‘type of preschool’ are dummy variables, with boys and public preschool as reference groups, respectively.

## Data Availability

Not applicable.
